# PATIENT PRIORITIES CARE FOR HISPANICS WITH DEMENTIA (PPC-HD)

**DOI:** 10.1093/geroni/igae098.0651

**Published:** 2024-12-31

**Authors:** Rafael Samper-Ternent

**Affiliations:** The University of Texas Health Houston, Houston, Texas, United States

## Abstract

Persons living with dementia frequently experience difficulty managing multiple chronic conditions (MCC) and have poorer outcomes. Managing MCC typically involves adhering to single-disease clinical practice guidelines. This approach results in burdensome care with outcomes that may not reflect what matters most to patients. Studies using the Patient Priorities Care (PPC) approach have demonstrated feasibility and effectiveness of PPC in different clinical settings. Physical, emotional, and cognitive impairments related to dementia interfere with disease self-management. Care partners, therefore, play an essential role in the care and decision-making of PlwD. Care partners are essential in translating ‘what matters most’ for PlwD into healthcare decisions. Patient and care partner involvement should include identifying outcome goals and care preferences (health priorities) as well as aligning care to meet those priorities. PlwD from minority groups face more barriers and have poorer outcomes compared to Non-Hispanic Whites. Hispanics are the fastest growing underrepresented population in the USA, and have higher risk of dementia compared to Non-Hispanic Whites. Hispanics rely heavily on their families and there is a cultural expectation of families to provide care to members in need. Cultural differences and language barriers shape healthcare priorities and how priorities impact outcomes among Hispanics. We recently completed a feasibility study among older Hispanics with cognitive impairment and their care partners. Results from this study confirm Hispanics with dementia and their care partners are willing and able to identify health priorities, and clinicians can align care to achieve these priorities when placed in the electronic health record (EHR).

